# The impact of a physician’s recommendation and gender on informed decision making: A randomized controlled study in a simulated decision situation

**DOI:** 10.1111/hex.13161

**Published:** 2020-12-04

**Authors:** Anna Lea Meinhardt, Marie Eggeling, Ulrike Cress, Joachim Kimmerle, Martina Bientzle

**Affiliations:** ^1^ Knowledge Construction Lab Leibniz‐Institut fuer Wissensmedien Tuebingen Germany; ^2^ Department of Psychology University of Tuebingen Tuebingen Germany

**Keywords:** attitude, decision satisfaction, gender, medical decision making, physiotherapy, recommendations, surgery

## Abstract

**Objective:**

This study examined the influence of physicians’ recommendations and gender on the decision‐making process in a preference‐sensitive situation.

**Methods:**

N = 201 participants were put in a hypothetical scenario in which they suffered from a rupture of the anterior cruciate ligament (ACL). They received general information on two equally successful treatment options for this injury (surgery vs physiotherapy) and answered questions regarding their treatment preference, certainty and satisfaction regarding their decision and attitude towards the treatment options. Then, participants watched a video that differed regarding physician's recommendation (surgery vs physiotherapy) and physician's gender (female vs male voice and picture). Afterwards, they indicated again their treatment preference, certainty, satisfaction and attitude, as well as the physician's professional and social competence.

**Results:**

Participants changed their treatment preferences in the direction of the physician's recommendation (*P* < .001). Decision certainty (*P* < .001) and satisfaction (*P* < .001) increased more strongly if the physician's recommendation was congruent with the participant's prior attitude than if the recommendation was contrary to the participant's prior attitude. Finally, participants’ attitudes towards the recommended treatment became more positive (surgery recommendation: *P* < .001; physiotherapy recommendation: *P* < .001). We found no influence of the physician's gender on participants’ decisions, attitudes, or competence assessments.

**Conclusion:**

This research indicates that physicians should be careful with recommendations when aiming for shared decisions, as they might influence patients even if the patients have been made aware that they should take their personal preferences into account. This could be particularly problematic if the recommendation is not in line with the patient's preferences.

## 1 INTRODUCTION

In many situations, patients have to make health‐related decisions. Such decisions are particularly challenging, especially when there are two or more treatment options which the empirical evidence confirms as equally effective. Decisions in such situations are referred to as preference‐sensitive, since in the end, the patient's preference will determine the decision made, and thus the individual needs and preferences of a patient must be taken into account very carefully.^1^, ^2^ However, many people find it difficult to make decisions about their own health.^3^, ^4^, ^5^ Often, medical expertise is needed to make an informed decision, or at least to indicate an informed preference for a treatment option.^6^, ^7^ Usually, patients are medical laypeople, and physicians often overestimate the medical knowledge of their patients.^8^ Many patients do not fully understand their physicians when receiving information about their condition, and they cannot remember information about treatments and risks adequately.^8^ Therefore, it is not enough just to give patients information; rather, decisions regarding their health should be discussed with the physician and made together.^9^, ^10^

Shared decision making (SDM) describes a decision‐making process in which physicians and patients are involved in the decision together. The partners in the decision‐making process should jointly discuss possible decision options, exchange relevant medical information and patient preferences and then decide on an option.^11^, ^12^ Although many studies show positive effects of SDM, there are still unresolved questions and ambivalent findings.^13^, ^14^, ^15^, ^16^, ^17^ The study presented here attempts to answer some of these still unresolved questions and aims to examine relevant effects.

The influence of a physician's recommendation for a treatment option has rarely been investigated.^18^, ^19^, ^20^ Only a few studies have investigated the influence of a recommendation in a preference‐sensitive decision situation (eg^21^, ^22^). As far as we know, there have been no studies yet that examined the influence of the physician's gender in the case of SDM. The study presented here is intended to contribute to understanding the effects of a physician's recommendation and gender in the case of a preference‐sensitive decision. The rupture of the anterior cruciate ligament (ACL) was selected as a preference‐sensitive situation.

### 1.1 Shared decision making

Fowler and colleagues showed that at the time of their study most medical decisions were made by physicians alone.^23^ However, most patients want to be involved in decision making and receive all relevant information—both positive and negative.^24^, ^25^ Therefore, it is not appropriate for physicians simply to inform their patients about a treatment decision they have already made.^26^

SDM goes beyond obtaining informed consent. The attending physician should not only obtain the consent of the patient, but also consider with the patient the risks, benefits and limitations of the different treatment options, as well as the patient's preferences; then, the physician and the patient should make a joint decision on a treatment option.^11^, ^12^, ^24^, ^27^, ^28^, ^29^ Charles and colleagues^11^ and Stiggelbout and colleagues^12^ described a four‐step model that defines SDM and provides physicians with a template for the process of joint decision making. The first step should be for the physician to inform the patient that a decision has to be made. In the second step, the various treatment options are named and explained, and their advantages and disadvantages described. In a third step, patients are then asked to pass on their wishes, values, preferences, and other important information to the physician. These are then discussed together. The collected information is considered and integrated into the treatment plan. In the fourth and final step, the patient's desired role in the decision‐making process is discussed, and a decision for treatment is taken jointly.

Previous research on SDM shows that this type of decision making has some advantages over mere informed consent. Many patients would like to make joint decisions about their treatment with their physician and prefer SDM over a physician‐centred decision.^24^ This is a trend that has increased in the years since 2000. In 71% of the studies in the years between 2000 and 2012, the people preferred SDM compared to 50% of studies before 2000.^24^

Stacey and colleagues found that SDM leads to greater patient confidence in the decision taken.^30^ Similarly, Shay and Lafata conclude that patients gain more knowledge, and attitude and emotions regarding the treatment are more positive if a joint decision has been made.^13^ SDM can also lead to less conflict and higher quality in decision making and a reduction in the frequency of operations^16^ and costs for the health‐care system.^31^, ^32^ Eggeling and colleagues have also found a reduction in decision‐making conflict.^21^ They simulated parts of the SDM process by showing their participants a video of a physician who explained all of the relevant information on treatment options and made it clear that the decision for a treatment option depended on their personal preferences. After the participants had received all of the relevant information about their treatment options, they stated that they experienced less conflict in making their decisions and were more satisfied with the decision‐making process, irrespective of the physician's recommendations.

### 1.2 Preference‐sensitive decisions

SDM can have a particularly positive effect on the decision‐making process in a preference‐sensitive decision situation.^21^, ^22^ A preference‐sensitive decision is one in which scientific evidence shows that no treatment option would have a better outcome than any other.^22^ This is the case, for example, with a rupture of the ACL.^33^, ^34^, ^35^, ^36^ For this injury, there are two equally promising treatment options. An ACL reconstruction is a surgical replacement of the ACL (surgical treatment option). The alternative treatment option is to have the musculature that surrounds the knee joint treated through physiotherapy. The musculature built up by the physiotherapy exercises is supposed to restore the stability of the knee even without the ACL and compensate for its absence (physiotherapeutic treatment option). There are cases in which surgical treatment cannot be avoided because the stability of the knee is too severely impaired.^33^, ^34^, ^37^ The decision situation can also differ for people who are highly active in sports. In particular, for professional athletes, this is not a preference‐sensitive decision since surgical intervention is usually more appropriate for people with a very athletic lifestyle, especially if they practice a sport that puts a strain on their knees. However, for the vast majority of people and if the stability of the knee is not so severely restricted that surgical intervention is unavoidable, both treatment options are comparably successful.^33^, ^34^, ^35^, ^36^, ^37^

A rupture of the ACL is therefore a preference‐sensitive decision situation and well suited for research purposes in the field of decision making in the medical context.^38^ Since no evidence definitively identifies one treatment option as better over any other, the personal experience and assessment of physicians and patients have a major impact on the choice of a treatment option. In this case, it is particularly important in the sense of SDM that both parties involved come to a decision jointly.

### 1.3 Physicians’ recommendations

Factors that may influence decisions in preference‐sensitive decision‐making situations are the physician's recommendation or lack of recommendation for one of the possible treatment options. The absence of a physician's recommendation and the resulting scientific uncertainty can lead to lower decision satisfaction and greater decision uncertainty.^39^ A physician's recommendation, on the other hand, can influence the decision for a treatment option even against the patient's previous preference, which also can subsequently lead to dissatisfaction with a decision.^19^ Scherr and colleagues found that a physician's recommendation can outweigh a patient's preference.^20^ They examined the influence of physicians’ recommendations on patients with prostate cancer. The study showed that the influence of the physician's recommendation exceeded the influence of the patient's preference. It also showed that in this case, the physician's recommendation was not based on the personal attitudes of the patients or the advantages and disadvantages of the different treatment options, but depended mainly on age and histological findings. The influence of physician's recommendations may even be so strong that patients choose a recommended treatment option even though this treatment is less promising for them.^18^

In a preference‐sensitive situation such as an ACL rupture, the physician's recommendation takes on even more importance, because both possible treatment options are promising, and the patient cannot purely rely on scientific evidence to make a treatment decision. Patients need to find additional reasons to make a decision. Since a physician's recommendation can result in less uncertainty and more satisfaction,^39^ following a physician's recommendation could lead to a reduction in the number of patients who are not satisfied with their treatment option in a preference‐sensitive situation. A recommendation that is in line with the patient's own preference should also lead to less conflict in decision making. Based on these considerations, we stated the following hypotheses that specified the impact of a physician's recommendation on participants’ treatment preferences, decision certainty and satisfaction, and their attitude towards the treatments.

Hypothesis 1 Participants who were recommended a surgical treatment show a higher preference for this treatment after the recommendation than participants who were recommended a physiotherapeutic treatment and vice versa.

Hypothesis 2 Decision certainty and satisfaction are lower for those participants who received a recommendation that was incongruent with their prior attitude than for those who received a recommendation that was congruent with their prior attitude.

Hypothesis 3 Participants who were recommended a surgical treatment show a more positive attitude towards this treatment than participants who were recommended a physiotherapeutic treatment (H3a). Participants who were recommended a physiotherapeutic treatment show a more positive attitude towards this treatment than participants who were recommended a surgical treatment (H3b).

### 1.4 Physicians’ gender

An aspect that influences adherence to a physician's recommendation is the patient's trust in the physician.^40^ The gender of the physician could affect this trust. It has been shown that female physicians were less trusted in training than their male counterparts.^41^ In addition, female physicians were given worse evaluations by patients than male physicians.^42^

Fassiotto and colleagues showed in their study that female physicians also received significantly worse ratings by other physicians in specialist training than their male colleagues. This was especially the case if the female physicians being evaluated worked in typically male‐dominated disciplines,^43^ including orthopedics and trauma surgery. These findings are relevant for the present study since these very disciplines would be involved in the ACL reconstruction surgery.

Research findings are largely unclear, however, regarding the question of how a physician's gender influences patients’ decisions. It is an empirically open question as to what extent a physician's gender has an impact in the context of SDM, especially in a preference‐sensitive situation. The present study seeks to answer this question. Based on the considerations, we stated the following hypotheses regarding the impact of a physician's gender on participants’ treatment preferences, attitude towards the treatments, and perception of professional competence.

Hypothesis 4 Participants who were recommended treatment by a male physician show a higher preference for this treatment after the recommendation than participants who were recommended treatment by a female physician.

Hypothesis 5 Participants who were recommended treatment by a male physician show a more positive attitude towards this treatment than participants who were recommended treatment by a female physician.

Hypothesis 6 Participants who received information and the recommendation from a male physician perceive their physician as more professionally competent than participants who received information and the recommendation from a female physician.

## 2 METHODS

We conducted a randomized controlled experiment in an online setting. The participants were placed in a hypothetical situation in which they had to imagine they suffered from an ACL rupture and had to decide on a treatment.

### 2.1 Ethical approval

The study presented here was part of a research project that was approved by the Ethics Committee of the Leibniz‐Institut für Wissensmedien.

### 2.2 Design

This study used a 2 (physician's recommendation: surgery vs physiotherapy) × 2 (physician's gender: female vs male) between‐groups design with repeated measurement. As dependent variables, we captured the participants’ treatment preference, the certainty and satisfaction regarding the decision, the attitude towards the treatment options and the assessment of the competence of the physician (professional and social). In addition, demographic data and the main reasons for choosing one of the treatment options were collected. The participants were randomly assigned to one of the four conditions. They were blinded to the other conditions. They were made aware of the purpose of the study and the other conditions only after participation.

### 2.3 Sample

**FIGURE 1:**
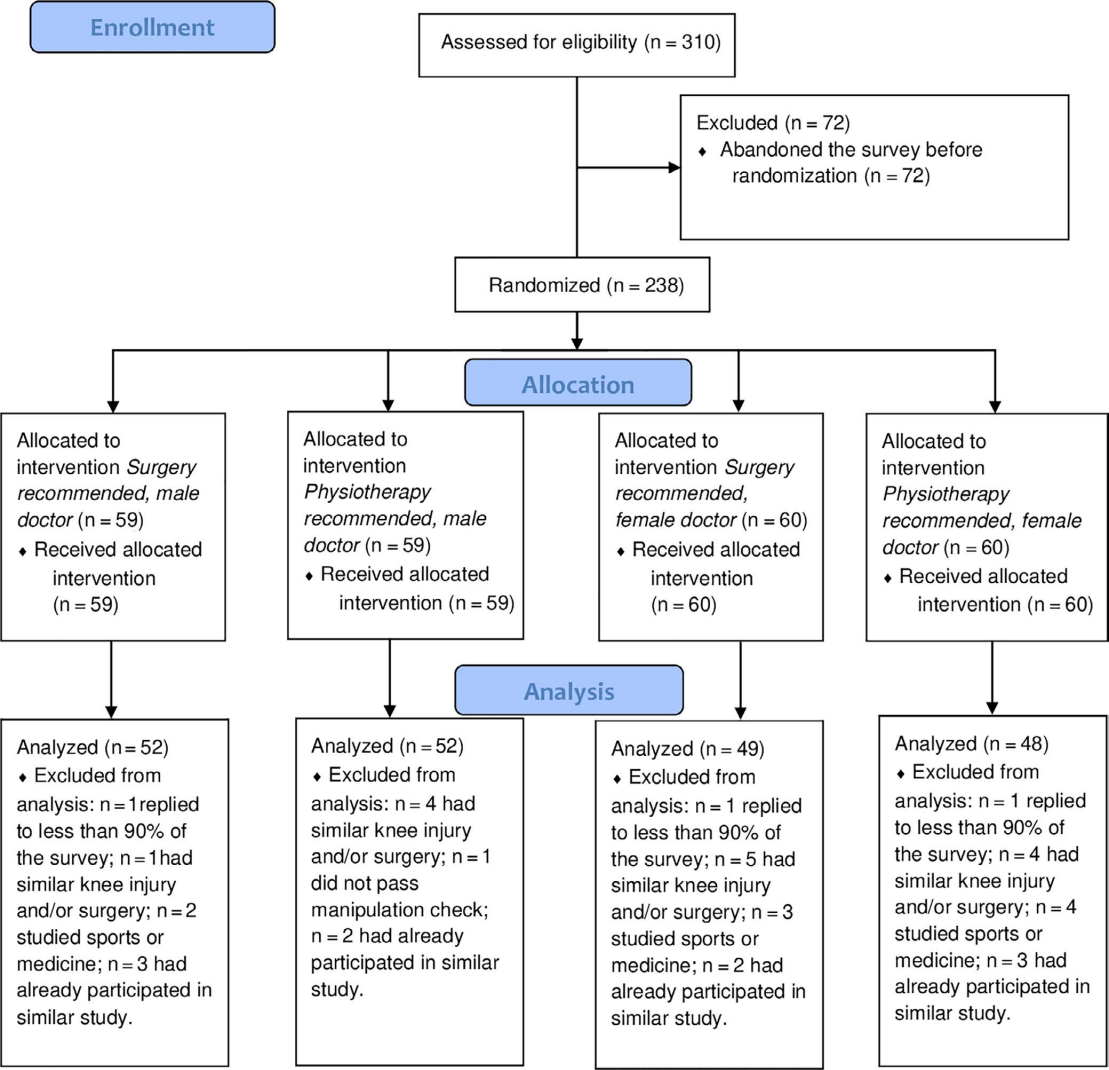
Sampling procedure

Power analysis for ANOVAs with α = 0.05, an intended power of 85%, and a medium size of f = 0.25 revealed a required sample size of N = 204. Because an ACL rupture often occurs among young and active people, we recruited mostly university students as participants. They were recruited via the e‐mail distribution list of the University of Tübingen. An invitation link to the study and information about the study were sent to potential participants via e‐mail. A total of 310 participants took part in the study. We excluded all participants who replied to <90% of the questionnaire. Since several properties could have a distorting effect on the study results, we excluded the following participants from the data analysis: (a) Participants who had already had an ACL rupture or a similar knee injury, (b) were students or professionals in the fields of medicine, sports science, or physiotherapy, (c) had participated in a previous study on that topic or (d) did not pass the manipulation check (see below). We analysed the data of the remaining 201 participants. The sampling procedure is shown in Figure 1.

One hundred ninety‐one participants were university students. Five participants were employees of the university. The remaining five participants either did not disclose their occupation (two) or worked in unrelated fields outside the university. The age of the participants was between 18 and 57 years old (mean 23.73, SD 4.88). One hundred forty‐nine participants identified as female, 50 as male and two as diverse. Participation in the study was voluntary, and all participants gave written informed consent. As a reward for participating in the study, they had the opportunity to take part in a raffle to win a voucher for an online shop.

### 2.4 Procedure

At the beginning of the online survey, participants were presented with information on the study and a declaration of consent. Demographic data such as age, gender, highest educational attainment and occupation were then collected. Subsequently, the hypothetical situation was described. Participants imagined that they had suffered an injury to their knee during sporting activity. While waiting for an MRI appointment to finally clarify whether the suspicion of an ACL rupture was confirmed, they should imagine that they were looking for information on the ACL and its rupture on the Internet.

Each group then received general information on the topic of ACL rupture. After reading this information, participants were asked to designate their treatment preference for surgery or physiotherapy, indicate their certainty and satisfaction regarding that decision, and rate their attitude towards the treatment options (Measurement t1).

After this first measurement, the participants were randomly assigned to one of four groups. All texts and questions were adapted to the respective conditions (ie to the gender of the physician). Participants were asked to put on headphones. They were shown a video in which either a drawing of a male or a female physician was shown as a still image. While the video was running, the participants listened either to a male or female voice who provided identical information in both conditions. During the simulated consultation, the physician confirmed the suspicion of an ACL rupture and provided further information on the diagnosis and the two possible treatment options. After the first part of the physician's talk, the participants listened to the second part of the talk, in which the physician gave them a recommendation for one of the treatment options.

Following the videos, the participants were asked for the second time (Measurement t2) which treatment option they would choose and to indicate certainty, satisfaction, and attitude. They were also asked to assess the professional and social competence of the physician and to specify the reasons that were most important to them personally for the decision‐making process.

Finally, a manipulation check was carried out. Participants were asked whether they could remember the gender of the advising physician and which recommendation was given. Participants who did not pass these test questions were excluded from further analyses (see above). Following the questionnaire, the participants were given the opportunity to enter their e‐mail address in a separate survey in order to take part in the draw for the vouchers.

### 2.5 Material

The questionnaire was created and administered using the online tool Qualtrics Survey Software.^44^ This Software enables the randomized assignment of participants to conditions, the incorporation of sound recordings, and the storage and export of anonymized research data to statistical analysis software. The audio recordings of the simulated consultations were recorded by a male and a female speaker in the sound studios of a public radio station. The scripts (Multimedia Appendix 1) and information texts (Multimedia Appendix 2) were taken from previous studies^21^, ^38^ and adapted for the research questions presented here.

### 2.6 Measures

In order to determine the treatment preference for one of the two possible treatment options, the participants answered a bipolar item ranging from 1 = surgery to 7 = physiotherapy.

Decision certainty was captured using the sub‐scale ‘decisional uncertainty’ (3 items) and decision satisfaction using the sub‐scale ‘perceived effective decision making’ (4 items) from the Decisional Conflict Scale.^45^ All items were measured on a seven‐point scale, with 1 = strongly disagree to 7 = strongly agree. Strong agreement indicated a high level of decision certainty and decision satisfaction. Both sub‐scales had good internal consistency. Decision certainty: Cronbach alpha at t1: *α* = 0.83; Cronbach alpha at t2: *α* = 0.76. Decision satisfaction: Cronbach alpha at t1: *α* = 0.88; Cronbach alpha at t2: *α* = 0.90. All items are shown in Table 1.

**Table 1:** Measurement of decision certainty and decision satisfaction

Decision certainty	Decision satisfaction
This decision is hard for me to make^a^	I feel I have made an informed choice
I’m unsure what to do in this decision^a^	My decision shows what is most important for me
It's clear what choice is best for me	I expect to stick with my decision
	I am satisfied with my decision

^a^ Indicates reversely coded items.

Attitudes towards treatment options were captured independently for each of the options with the seven‐point scale by Marteau and colleagues.^46^ Attitude towards surgery: Cronbach alpha at t1: *α* = 0.77; Cronbach alpha at t2: *α* = 0.81. Attitude towards physiotherapy: Cronbach alpha at t1: *α* = 0.80; Cronbach alpha at t2: *α* = 0.83. The four items of this scale are shown in Table 2.

**Table 2:** Measurement of attitude towards the treatments

For me, surgery/physiotherapy after a rupture of the anterior cruciate ligament would be…
(1) beneficial – (7) harmful^a^
(1) important – (7) unimportant^a^
(1) a bad thing – (7) a good thing
(1) unpleasant – (7) pleasant

^a^ Indicates reversely coded items.

The professional and social competence of the physician (Cronbach alpha social competence: *α* = 0.87; Cronbach alpha professional competence *α* = 0.89) was measured using the nine‐level Perceived Professional and Social Competence scale by Willson and McNamara.^47^ The 17 items of this scale are shown in Table 3.

**Table 3:** Measurement of professional and social competence

Professional competence	Social competence
(1) Unprofessional – (9) Professional	(1) Friendly – (9) Unfriendly^a^
(1) Experienced – (9) Inexperienced^a^	(1) Impolite – (9) Polite
(1) Not thorough – (9) Thorough	(1) Attentive – (9) Not attentive^a^
(1) Careful – (9) Careless^a^	(1) Unkind – (9) Kind
(1) Incompetent – (9) Competent	(1) Pleasant – (9) Unpleasant^a^
(1) Trained – (9) Untrained^a^	(1) Not nice – (9) Nice
(1) Not appealing – (9) Appealing	(1) Caring – (9) Not caring^a^
(1) Confident – (9) Unconfident^a^	(1) Insensitive– (9) Sensitive
	(1) Sympathetic – (9) Unsympathetic^a^

^a^ Indicates reversely coded items.

Finally, the reasons for the participants’ decision in favour of a treatment option were asked using a single‐choice question. Participants indicated the most important factor for their decision from a list with five options: prior personal experience with the topic, the recommendation of the physician, information text about the treatment options, information gained from the medical consultation or other.

### 2.7 Analysis

Data analysis was performed using IBM SPSS 25 statistics for Windows. Normal distribution was not given for most variables. We performed 2‐factorial or 1‐factorial (M)ANOVAs for all hypotheses except Hypothesis 2, because simulation studies have shown that ANOVAs are robust to violations of the normal distribution assumption.^48^, ^49^ We provide means and standard deviations (SD) as well as *F*‐values, *P*‐values and partial eta‐squared (_part._ η^2^) as an indicator of effect size.

Hypothesis 2 was tested using a two‐way interaction linear regression analysis, followed by a simple slope analysis. We provide regression coefficient B, standard error SE and *P*‐values.

The significance level for all analyses was set to *α* = 0.05.

## 3 RESULTS

Before receiving a recommendation from the physician, participants’ treatment preference did not differ among the conditions, *P* = .662. In Hypothesis 1, we had stated an impact of the physician's recommendation on participants’ treatment preferences. The data supported this hypothesis. Participants who were recommended a surgical treatment showed a higher preference for this treatment after the recommendation (mean 3.51, SD 2.04) than participants who were recommended a physiotherapeutic treatment (mean 5.64, SD 1.72), F(1, 199) = 64.36, *P* < .001, _part._ η^2^ = 0.24.

**FIGURE 2:**
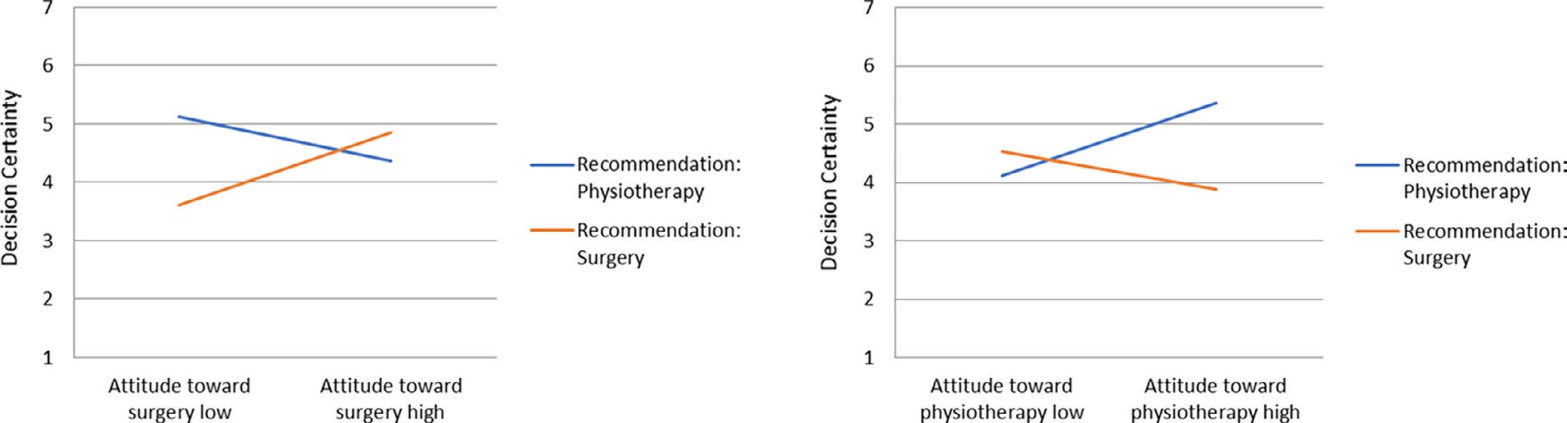
Linear regression analysis for decision certainty

**FIGURE 3:**
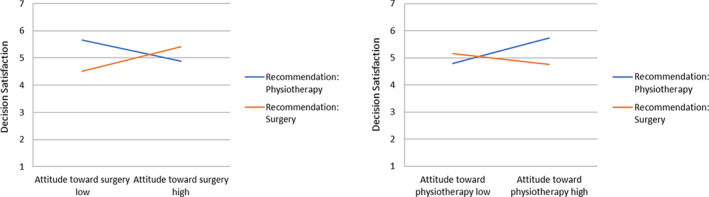
Linear regression analysis for decision satisfaction

In Hypothesis 2, we had stated the impact of the physician's recommendations on decision certainty and satisfaction. We assumed that certainty and satisfaction would be higher for those participants who received a recommendation that was congruent with their prior attitude. To test this assumption, we calculated a linear regression analysis with physician's recommendation × prior attitude towards surgical treatment and physician's recommendation × prior attitude towards physiotherapy as interaction terms; the predicted interaction reached significance for all regression models for both decision certainty and satisfaction (see Table 4 and Figures 2 and 3).

**Table 4:** Effects of linear regression analysis with physician's recommendation × prior attitude towards surgical treatment and physician's recommendation × prior attitude towards physiotherapy as interaction terms

	Decision certainty			Decision satisfaction		
	B	SE	*P*	B	SE	*P*
Physician's recommendation × prior attitude towards surgical treatment	0.50	0.10	<.001	0.42	0.09	<.001
Physician's recommendation × prior attitude towards physiotherapy	−0.47	0.10	<.001	−0.33	0.09	<.001

In line with our hypothesis, simple slope analyses showed that for participants who received a recommendation for surgery, relatively positive attitudes towards surgery predicted high certainty (B = 0.62, SE = 0.15, *P* < .001) and satisfaction (B = 0.45, SE = 0.13, *P* = .001) with their decision. For participants who received a recommendation for physiotherapy, relatively positive attitudes towards physiotherapy predicted high certainty (B = 0.62, SE = 0.15, *P* < .001) and satisfaction (B = 0.47, SE = 0.13, *P* < .001) with their decision (congruency effect).

Interestingly, we found nearly the same pattern also the other way around: For participants who received a recommendation for surgery, relatively positive attitudes towards physiotherapy predicted low certainty (B = −0.32, SE = 0.14, *P* = .021). Low satisfaction with their decision, however, was not predicted by positives attitudes towards physiotherapy (*P* = .106). For participants who received a recommendation for physiotherapy, relatively positive attitudes towards surgery predicted low certainty (B = −0.37, SE = 0.14, *P* = .007) and low satisfaction (B = −0.39, SE = 0.12, *P* = .001) with their decision (incongruency effect).

In Hypothesis 3, we had stated an impact of the physician's recommendation on participants’ attitudes towards surgery (H3a) and physiotherapy (H3b). Before receiving a recommendation, participants’ attitudes towards surgery (*P* = .782) and physiotherapy (*P* = .902) did not differ among the conditions. Supporting H3a, participants who were recommended a surgical treatment showed a more positive attitude towards surgery (mean 4.35, SD 1.17) than participants who were recommended a physiotherapeutic treatment (mean 3.39, SD 1.17), F(1, 199) = 34.14, *P* < .001, _part._ η^2^ = 0.15. Supporting H3b, participants who were recommended physiotherapy showed a more positive attitude towards physiotherapy (mean 5.22, SD 1.11) than participants who were recommended surgery (mean 4.57, SD 1.24), F(1, 199) = 15.40, *P* < .001, _part._ η^2^ = 0.07.

In Hypothesis 4, we had stated an impact of the physician's gender on participants’ treatment preferences. Contrary to this hypothesis, there was no difference between participants who had received a recommendation by a male physician (mean 4.62, SD 2.14) and participants who had received a recommendation by a female physician (mean 4.52, SD 2.20), F(1, 199) = 0.11, *P* = .745.

In Hypothesis 5, we had stated an impact of the physician's gender on participants’ attitudes towards the recommended treatment. Our analysis showed no significant interaction effect between physician's gender and recommendation on participants’ attitudes towards surgery, F(1, 197) = 0.23, *P* = .631, and physiotherapy, F(1, 197) = 0.50, *P *= .479. This means that contrary to this hypothesis, there was no difference between participants who had received a recommendation by a male physician (attitude surgery: mean 3.92, SD 1.19; attitude physiotherapy: mean 4.83, SD 1.18) and participants who had received a recommendation by a female physician (attitude surgery: mean 3.83, SD 1.34; attitude physiotherapy: mean 4.95, SD 1.26).

In Hypothesis 6, we had stated an impact of the physician's gender on participants’ perception of the physician's professional competence. Contrary to this hypothesis, we found no difference between participants who had received a recommendation by a male physician (mean 7.38, SD 1.09) and participants who had received a recommendation by a female physician (mean 7.40, SD 1.21), F(1, 199) = 0.03, *P* = .855. We also found no difference in the assessment of social competence, F(1, 199) = 0.15, *P* = .703.

As an exploratory analysis, we captured the most important reasons for the participants’ decision in favour of a treatment option. Seventy‐six participants (37.8%) rated the information they gained from the simulated consultation as the most important. Sixty‐one (30.3%) participants indicated the recommendation of the physician as the most important reason. Thirty‐four participants (16.9%) named prior personal experience with the topic, and 15 participants (7.5%) mentioned the information text about the treatment options as most important. Thirteen participants (6.5%) marked other reasons and mentioned reasons like fear of surgery or duration of therapy.

## 4 DISCUSSION

This study examined the influence of a physician's recommendation and gender on the decision‐making process in a preference‐sensitive decision‐making situation. While the data strongly supported the hypotheses regarding the influence of a physician's recommendation, the hypotheses on the influence of a physician's gender were all rejected. We found an influence of the physician's recommendation on the treatment preference, decision certainty and satisfaction, and the attitude towards the possible treatment options. Although the physician had expressly stated in the study video that the decision for a treatment option should be based solely on the preference of the participants, the physician's recommendation had an evident impact. About 30 per cent of the participants explicitly mentioned the physician's recommendation as the most important reason for their decision. Since this was a preference‐sensitive decision where two treatment options showed equally good treatment outcomes, this influence is particularly interesting. The present study also found a reduction in decision conflict: If the physician's recommendation corresponded with the participants’ attitude, decision certainty and satisfaction increased significantly more strongly than with an incongruent recommendation. Decision certainty and satisfaction were higher if the physician's recommendation corresponded to the participants’ attitude and lower if the recommendation was incongruent with participants’ attitude.

These results are particularly interesting for physicians who aim to share decision making with their patients. While the study shows that a physician's recommendation seems to have a significant influence on the patients’ decision, this is not in the spirit of shared decision making, where patients and their preferences should be a priority.^12^ Especially in the case of a preference‐sensitive decision situation in which two treatment options show a comparable recovery success, the recommendation of the physician should not be the key factor for the decision of the patients. Physicians should be aware that their recommendations not only have a substantial impact but that patients may also assume that the physician's recommendation was made based on information they were not given. So, if physicians want to make a shared decision with their patients, they should not only be careful with their recommendation but also ensure that they disclose the reasons and motives for their recommendation. In addition, it could be detrimental if the recommendation does not correspond to the patient's attitude. It is therefore essential that a physician be aware of the patient's attitudes and preferences. As it would be good for patients to feel as confident and satisfied with their decision as possible, it might be beneficial for physicians, who wish to establish shared decision making, not to make a treatment recommendation at all. Alternatively, physicians could wait and see until they know the preference of their patients and only then decide whether to make a recommendation or not. Future research should investigate how physicians cope with this type of conversation and under what circumstances they can adapt their communication style accordingly. This research could also examine physicians’ and patients’ understanding of their own roles and analyse their expectations of their conversation partner.

Contrary to our expectation that participants follow the recommendation of a female physician less often than that of a male physician, the study showed that gender did not influence participants’ decisions in favour of a treatment option. Nor was there any influence of the physician's gender on the change of attitude and the perceived professional or social competence. The comparison with the study by Bonds and colleagues,^41^ which investigated the extent to which patients trusted physicians, is interesting. In their study, trust in physicians was relatively high, but trust was positively associated with male physicians. Differences between those findings and the present study could be due to the different samples. The participants of Bonds and colleagues^41^ were actual patients in a hospital, their average age was much higher (mean 54 years), and their educational level lower. Only 25% of the patients had some kind of academic education. The participants in the present study were younger and more highly educated. Therefore, the question arises whether age and educational level might have influenced the findings on gender stereotypes. This question should be further investigated in future studies that explicitly control for age and educational level. Moreover, the study by Bonds and colleagues^41^ was published in 2004 and it is possible that gender stereotypes have changed since then. More recent studies from other medical domains have found only small^50^ or no gender differences.^51^ Further research should systematically investigate in which health‐related areas problematic gender stereotypes still prevail and how they can be addressed.

As a meta‐analysis has shown, ‘(f)emale primary care physicians engage in more communication that can be considered patient centered … than their male colleagues.’ (p. 756,^52^). In our study, the male and female physician used the exact same communication style. In future studies, it could be investigated if there is an interaction effect of gender and communication style on the evaluation of physicians. It is plausible that the communicative behaviour of a physician has a stronger influence than gender itself.^53^, ^54^

Overall, we consider the results of the present study to be quite encouraging from the perspective of gender‐equality initiatives. Since neither the participants’ decisions, attitudes nor their perception of competence were dependent on the physician's gender, this allows for the conclusion that the sample tested did not judge people or their information based on gender.

Despite the insights that this study provides, it also has some limitations. This includes the fact that the participants did not have a real physician‐patient conversation, but only watched a simulated video. Accordingly, the consultation did not include interactive elements. It is not possible to conclusively evaluate the extent to which people would have behaved differently in a real consultation. Moreover, the participants were healthy people who were only supposed to imagine their injury and not real patients, and it cannot be said how well the participants managed to put themselves in this situation. Future research should therefore aim at replicating these findings with real patients who are in a real conversation situation with a physician. In addition, we had a rather particular sample of participants, which contained a disproportionate number of females. This high number of female participants was due to the recruitment process using the e‐mail distribution list of a university that offers numerous humanities courses with a high proportion of women. Further research with a more balanced sample is necessary. Finally, we only investigated treatment preferences and attitudes towards treatments and did not record any real decision‐making behaviour. These aspects should be considered more thoroughly in further studies with real patients.

## 5 CONCLUSION

This study contributed some new findings on whether a physician's recommendation and gender influenced the decision‐making process in a preference‐sensitive decision‐making situation. Our findings show the influence of a physician's recommendation on the treatment preference, decision certainty and satisfaction, and the attitude towards the recommended treatment. The study did not reveal any gender effects, indicating that there was no preference for one gender, and there was no evidence of discriminative behaviour in response to a male or female physician.

## 6 PRACTICE IMPLICATIONS

Our findings indicate that physicians should be careful with recommendations when aiming for shared decisions, as they might influence patients even if the patients have been made aware that they should give weight to their personal preferences. This could be particularly problematic if the recommendation is not in line with prior attitudes, as this lessens a patient's certainty and satisfaction regarding the decision. Physicians should always be aware of the effect of their recommendations and be mindful of this influence in the conversation situation.

## CONFLICT OF INTEREST

No conflicts of interest are declared.

## AUTHORS’ CONTRIBUTION

All authors contributed substantially to the conception and design of this work. AM was involved in the acquisition of data, AM, ME and MB analysed and interpreted the data; all the authors contributed significantly to the discussion. JK, MB and ME drafted the manuscript; UC and AM commented on it and critically revised it. All of the authors approved to the final version to be published; all of the authors agreed to be accountable for all aspects of the work in ensuring that questions related to the accuracy or integrity of any part of the work would be appropriately investigated and resolved.

## PATIENT OF PUBLIC CONTRIBUTION

There were no patients, service‐users or care‐givers involved in our study. The participants in our study were mostly university students who took part in an online experiment.

## Data Availability

The data that support the findings of this study are available from osf.io/y467e.^55^

## Appendix 1 Hypothetical situation and video script

### Part 1

Imagine you are in the following situation

In your free time you like to do sports. A few days ago, you had an accident while doing sports: During a suboptimal movement you twisted your left knee. At first you experienced great pain, which was relieved slightly by cooling. Your knee has become very swollen since then and you have difficulties walking.

With the injury you go to your doctor and tell about the accident and the symptoms that you have since. The doctor examines your knee externally and tells you that the injury is possibly a rupture of the anterior cruciate ligament. However, this cannot be said exactly at the moment, because the knee is very swollen. Therefore, the doctor refers you to the radiologist to have a magnetic resonance imaging (MRI) performed and to clarify if your cruciate ligament is actually torn. In the meantime, you should protect your knee and get crutches for support.

At home, you arrange an MRI‐appointment for 3 weeks later. To learn more about the cruciate ligament and cruciate ligament injuries, you search the web for information. There you find the following information:

### Part 2

A few weeks later, the swelling in your knee has subsided significantly. You hardly have pain anymore and you can already walk better. Only sometimes the knee still feels a bit shaky. You keep your MRI appointment as planned. Since the results of the MRI ware sent to your doctor by the radiologist, you see your doctor to discuss them together.

Now you hear two parts of the conversation with your doctor. Please listen carefully until the end. Please do NOT pause in between.

Please put on HEADPHONES now or turn on your SPEAKERS.

### Introduction for everyone

Good afternoon. I heard that your knee is feeling better and you can treat again without pain. I’m glad to hear that.

It's good that you had the MRI scan anyway, because unfortunately my presumption was confirmed: Your anterior cruciate ligament is torn. Fortunately, it looks like there are no other injuries.

Thanks to the diagnosis, we can now discuss your treatment options. There are two different treatment options for a cruciate ligament rupture. A cruciate ligament rupture can be treated both surgically and non‐surgically.

During surgical treatment, the broken anterior cruciate ligament is replaced by a transplant, made of an endogenous tendon. The surgery usually takes place stationary and under general anesthesia. It lasts about one to one and a half hours. The procedure is performed arthroscopically. In arthroscopic surgery only a few, several millimeters long surgical cuts are made, the surgery is possible without major, externally visible wounds or scars. The transplant is usually created from one or more endogenous tendons, for example the semitendinosus tendon. In the weeks and months after the surgery, post‐treatment physiotherapy is prescribed, which is very important to restore a proper function of the knee joint.

During non‐surgical treatment, the knee is trained extensively in physiotherapy. Due to the lack of movement through the protection of the knee joint, the knee‐surrounding muscles severely degraded. The aim is that it will soon be possible again to completely stretch and bend the knee. Once this is achieved, the muscle growth and the training of the muscle control through strength and stability training are paramount. Later, protective reflexes are trained through coordination exercises and reactive force training. The central goal of the non‐surgical treatment is to train the muscles so, thus compensating the stability lost by the cruciate ligament injury. The non‐surgical treatment should begin as soon as possible and takes several months. If the physiotherapy is not enough because the knee remains too unstable, surgery can be performed later. As I said, good results are possible with both treatments and you should think carefully about which treatment you prefer for yourself.

### Recommendation for physiotherapy

I would recommend that you first try the non‐surgical treatment. In a study comparing both treatment methods, it was found that 5 years after the injury most of the patients who opted for physiotherapy were satisfied with the outcome and did not need surgery. So you have a good chance of avoiding a surgical procedure that in itself is a burden for the body and involves risks. For some cruciate ligament surgeries, there are complications such as infections, thrombosis, non‐ingrowth of the graft. Only recently, we performed surgery on a person of your age and the graft didn't grow in properly, so the surgery had to be done a second time.

The disadvantage of the non‐surgical treatment is that despite physiotherapy the instability of the knee could remain too strong, which would mean that in a few months you would need surgery anyway. In this case, the treatment would take a little longer overall.

Another scientific study has shown that some of the patients who had surgery needed to have another surgery because in the following years they had a rupture of the replacement plastic. Although it happens regularly that surgically‐treated patients show a higher stability in physiological tests, they usually report no differences regarding the personal perception of stability. Physiological tests measure the isolated stability and certain movements, the personal perception refers to the assessment of function and stability of the knee in everyday life. In addition, there is no difference in the occurrence of early osteoarthritis between patients who were treated surgically and non‐surgically. For these reasons, I recommend you try the non‐surgical treatment first.

### Recommendation for surgery

I would recommend that you have surgery immediately. In a study comparing surgical and non‐surgical treatment methods, it was found that 5 years after the injury most of the patients who opted for surgery were satisfied with their outcome. Of course, a surgical procedure is always a burden for the body and involves risks. For some cruciate ligament surgeries, there are complications such as infections, thrombosis, non‐ingrowth of the graft. But the risk is low, I have experienced such complications with only very few people of your age.

Although you may be able to avoid surgery by having physiotherapy, there is always the risk that despite physiotherapy the instability of the knee could remain too strong, which would mean that in a few months you would need surgery anyway. It is common that patients who were treated non‐surgically report no differences regarding the personal perception of stability, but surgically‐treated patients usually show a higher stability in physiological tests. Physiological tests measure the isolated stability and certain movements, the personal perception refers to the assessment of function and stability of the knee in everyday life. In addition, there is no difference in the occurrence of early osteoarthritis between patients who were treated surgically and non‐surgically. For these reasons, I recommend you undergo surgery.

## Appendix 2 General information text

### What is the cruciate ligament and what do we need it for?

The knee joint is stabilized by a number of ligaments. The cruciate ligament is part of this ligamentous apparatus. Without the cruciate ligaments, the knee would be very unstable. There are anterior and posterior cruciate ligaments, which intersect in the centre of the joint—hence the name ‘cruciate ligament’. They connect thighs and shins and guide the knee in every movement.

Despite their high resilience, the cruciate ligaments are susceptible to injuries. Rupture of the cruciate ligament is one of the most common and serious sports injuries, with more than 90% of cases affecting the anterior cruciate ligament. There is an especially great danger during skiing as well as ball sports like football or basketball. The anterior cruciate ligament tear is often a sports injury, which occurs by twisting the lower leg away from its natural mechanical axis (= sprain).

Most sufferers of a cruciate ligament injury feel a sharp, stabbing pain and some hear a crack at the moment of the tear. Typical symptoms include swelling of the knee joint, instability of the knee, problems with stretching and bending the knee, and bruising in the knee area. The severity of these symptoms and the associated pain can vary significantly.

Recognizing a cruciate ligament tear is not easy for doctors, as the cruciate ligaments lie deep in the knee joint. Thus, it often happens that a diagnosis takes weeks or even years. Magnetic resonance imaging (MRI) or arthroscopy usually makes it possible to diagnose the injury. Knee Osteoarthritis can occur as a late consequence of a cruciate ligament tear.

### Treatment options

After a tear of the anterior cruciate ligament, there is the possibility to perform an operation in which the injured cruciate ligament is replaced by a transplant, or to treat conservatively, that is to strengthen the knee with the help of physiotherapy. Neither option is clearly preferable given the current scientific situation and both methods can lead to good treatment outcomes (Meuffels et al, 2009; Streich et al, 2011, Monk et al, 2016).
